# Role of microenvironment characteristics and MRI radiomics in the risk stratification of distant metastases in rectal cancer: a diagnostic study

**DOI:** 10.1097/JS9.0000000000001916

**Published:** 2024-09-04

**Authors:** Qing Zhao, Hongxia Zhong, Xu Guan, Lijuan Wan, Xinming Zhao, Shuangmei Zou, Hongmei Zhang

**Affiliations:** a Department of Diagnostic Radiology, National Cancer Center/National Clinical Research Center for Cancer/Cancer Hospital, Chinese Academy of Medical Sciences and Peking Union Medical College; b Department of Pathology, National Cancer Center/National Clinical Research Center for Cancer/Cancer Hospital, Chinese Academy of Medical Sciences and Peking Union Medical College; c Department of Colorectal Surgery, National Cancer Center/National Clinical Research Center for Cancer/Cancer Hospital, Chinese Academy of Medical Sciences and Peking Union Medical College, Beijing; d Department of Radiology, Shanxi Province Cancer Hospital/Shanxi Hospital Affiliated to Cancer Hospital, Chinese Academy of Medical Sciences/Cancer Hospital Affiliated to Shanxi Medical University, Taiyuan, People’s Republic of China

**Keywords:** distant metastases, magnetic resonance imaging, radiomics, rectal cancer, tumor stromal ratio

## Abstract

**Objectives:**

To compare the value of tumor stroma ratio (TSR) and radiomic signature from baseline MRI for stratifying the risk of distant metastases (DM) in patients with locally advanced rectal cancer (LARC).

**Materials and methods:**

Data from 302 patients with LARC who underwent neoadjuvant chemoradiotherapy and total mesorectal excision in our hospital between 2015 and 2018 were retrospectively reviewed, and the patients were randomly allocated into the training and validation cohorts in a ratio of 7:3. Patients were followed-up for more than 3 years postoperatively with metachronous DM as the endpoint. Independent risk factors for DM-free survival (DMFS) were analyzed using Cox regression. The TSR of endoscopic biopsy specimens was scored automatically. Totally 1229 radiomic features of each tumor were extracted from baseline MRI, and the Radscore was calculated.

**Results:**

The median follow-up time was 54.3 (51.6–57.1) months, and the 3-year DMFS was 83.8%. The best cutoff value of the TSR to distinguish a patient’s DM risk was 0.477 (Sen=70.8%, Sep=78%, *P*<0.001). Increased TSR (HR=3.072, *P*=0.006) and Radscore (HR=719.231, *P*=0.023), advanced MR-evaluated T stage (HR=2.660, *P*=0.023) and ypN (HR=2.362, *P*=0.028) stage were independent risk factors for DMFS. The area under the curve of the combined model was significantly higher than that of the radiomic model (*P*=0.013) but without a significant advantage over the TSR model (*P*=0.086).

**Conclusion:**

TSR of colonoscopic biopsies can independently stratify DM risk in patients with LARC. The TSR model is the most convenient and efficient method for DM risk stratification in LARC.

## Graphical abstract

**Figure:**
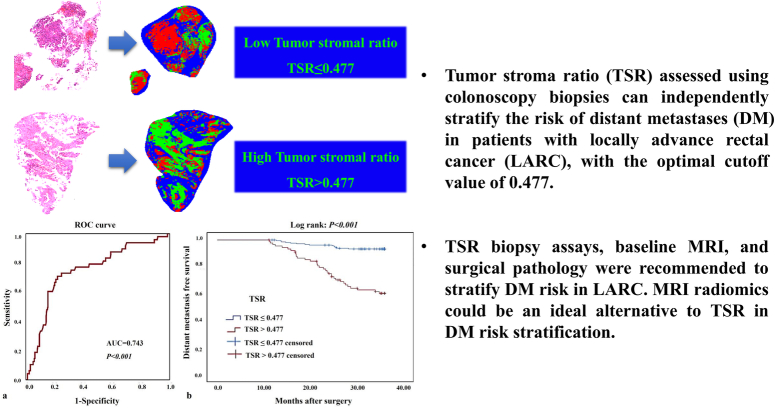

## Introduction

Highlights

- Tumor stroma ratio (TSR) of biopsies significantly affects distant metastases (DM) risk in LARC.
- TSR biopsy assay, baseline MRI, and surgical pathology are highly recommended for DM risk stratification.
- In patients unavailable for colonoscopy biopsy, MRI radiomic analysis could be an ideal alternative.

Rectal cancer (RC) incidence is increasing annually in China^1^. Early studies^2^ and our preliminary research^3^ have shown that neoadjuvant chemoradiotherapy (NCRT) can significantly improve disease-free survival in locally advanced rectal cancer (LARC). Approximately 17–25% of these patients still develop distant metastases (DM) after NCRT and radical excision^4,5^, which is the leading cause of RC-related death^6^. Accurate stratification of patients’ risk of developing metachronous DM to guide individualized management and follow-up is thus an urgent clinical issue.

MRI is the recommended method for evaluating the local stage and risk factors of RC^7^, from which radiomic studies can reveal more heterogeneous information about the tumor. Radiomic features significantly correlate with tumor invasiveness, treatment response, and patient outcomes^8^. However, the efficacy of radiomic methods in predicting DM risk remains limited, and the complexity, instability, and weak interpretability of the features limit their clinical application.

The tumor stroma mainly comprises mesenchymal cells and the extracellular matrix, constituting the most critical components of the tumor microenvironment and the main source of tumor heterogeneity. It plays a vital role in tumor cells’ growth, invasion, migration, and immune evasion^9^. The tumor-stromal ratio (TSR), reflecting the proportion of stromal components in the entire tumor tissue^10^, is an important characteristic of the tumor microenvironment and has emerged as a valuable prognostic factor for many malignancies, including RC^11^. The current recognized TSR measurement is based on analyzing paraffin sections stained with eosin and hematoxylin at the front of the invasive tumor that requires complete primary excision. However, for LARC, tremendous changes may occur in the tumor microenvironment after NCRT. Additionally, in patients deemed ineligible for surgery, TSR cannot be measured.

To resolve the drawbacks and fully explore the influence of TSR on the DM outcomes of RC, we hypothesized that TSR assessed using endoscopic biopsies before NCRT can partially reflect the tumor heterogeneity and affect patient survival. Therefore, in this study, we aimed to investigate the relationship between the biopsic TSR and metachronous DM risk in LARC patients treated with NCRT and radical resection. Additionally, we aimed to compare the value of tumor microenvironment characteristics represented by TSR and MRI radiomics in stratifying DM risk and to construct a prediction model with the most convenient clinical application and efficiency.

## Materials and methods

### Patients

This study was retrospectively registered and was approved by the review board of our institute, with a waived informed consent (22/449-3651). We retrospectively reviewed consecutive cases of rectal adenocarcinoma who were treated with 5-FU or Capecitabine-based concurrent NCRT and total mesorectal excision at our institute between 2015 and 2018. This retrospective cohort study was registered with the ClinicalTrials. The work has been reported in line with the STARD (Standards for the Reporting of Diagnostic Accuracy Studies) criteria^12^ (Supplemental Digital Content 1, http://links.lww.com/JS9/D383).

**Figure 1:**
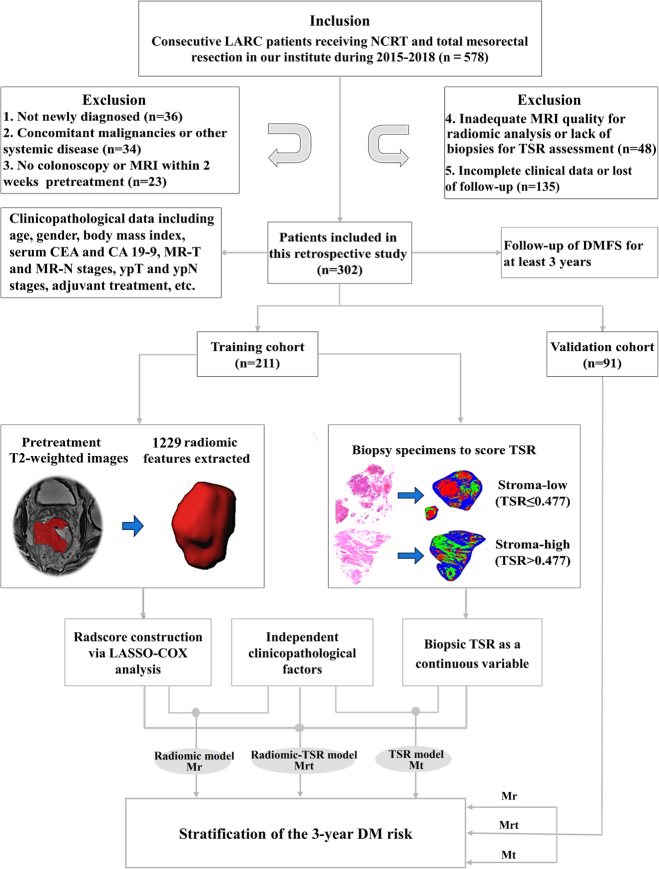
Study flowchart. CEA, carcinoembryonic antigen; CA 19-9, cancer antigen19-9; DMFS, distant metastasis-free survival; LARC, locally advanced rectal cancer; Mr, radiomic model; Mt, TSR model; Mrt, radiomic-TSR model; NCRT, neoadjuvant chemoradiotherapy; TSR, tumor stroma ratio.

The inclusion criteria were: (1) newly diagnosed non-mucinous LARC (cT_3_N_0_M_0_, cT_4_N_0_M_0_, or any T with N_1~2_M_0_) without previous treatment; (2) No concomitant malignancies or systemic disease; (3) complete NCRT and radical surgery in our institute; (4) underwent rectal MRI and colonoscopy within 2 weeks before NCRT. The exclusion criteria were: (1) fail to meet any of the inclusion criteria; (2) inadequate MR image quality for radiomic analysis, or lack of biopsy tissue for TSR assessment; (3) incomplete clinical data or withdraw before the last visit. A total of 578 eligible LARC cases were initially reviewed, 276 of which were excluded according to the exclusion criteria. Finally, 302 patients were enrolled in this study and randomized into a training cohort (*n*=211) and a validation cohort (*n*=91) (Fig. 1).

The patients’ clinicopathological data were reviewed, including age, sex, BMI, serum carcinoembryonic antigen (CEA) and cancer antigen (CA) 19-9 levels, T-stages and N-stages evaluated by MRI, yp-T, and yp-N stages evaluated by histopathology. The application of adjuvant therapy was recorded. The MR-T/N stages of the tumors were assessed by two experienced radiologists with 10 (Z.Q.) and 28 (Z.H.M.) years of experience on a picture-archiving and communication system workstation, with any disagreement resolved by consensus.

### Follow-up

Postoperative follow-up was arranged quarterly for the first year and semiannually for at least 3 years thereafter for all patients. DM was defined as cancer recurrence at sites outside the pelvis based on radiological and/or histopathological evidence. Distant metastasis-free survival (DMFS) was calculated from the date of surgery to the date of the first detection of DM, death from any cause, or last follow-up.

### TSR evaluation

Biopsy specimens from colonoscopy were sectioned into 5 μm slices and stained with eosin and hematoxylin. Areas with both stromal and tumor cells presented on all four sides were selected to evaluate TSR using an automated scoring method. The highest proportion of stromal components in all measured areas was recorded as the final TSR value in this study.

### MR technique

Pretreatment MR examinations were conducted using 3.0 T scanners with an 8-channel phased-array wrap-around surface coil. An intramuscular injection of 10 mg raceanisodamine hydrochloride was administered to minimize bowel movement unless contraindicated. Sequences acquired included T1-weighted imaging (T1WI), T2-weighted imaging (T2WI) with and without fat saturation, and diffusion-weighted imaging (DWI) (Supplemental Digital Content 2, http://links.lww.com/JS9/D384).

### Radiomic study

#### Tumor segmentation

The tumor region of interest was manually delineated slice-by-slice on high-resolution oblique axial T2WI (orthogonal to the rectal lumen) by the first radiologist with 5 years of experience (Z.H.X.) and subsequently confirmed by the second radiologist with 10 years of experience (Z.Q.) on ITK-SNAP, from which the three-dimensional whole tumor volume of interest (VOI) were obtained. Disagreements were resolved through discussions. The radiologists were blinded to the clinicopathological information.

#### Radiomic feature extraction and selection

PyRadiomics was used for image preprocessing and feature extraction. Overall, 1229 radiomic features (Supplemental Digital Content 3, http://links.lww.com/JS9/D385) were extracted from each VOI, which can be classified into four categories: (1) shape characteristics; (2) first-order statistical characteristics; (3) texture features; and (4) high-order statistical characteristics.

The extracted features’ interclass and intraclass correlation coefficients (ICCs) were calculated to assess the reproducibility of the radiomic features. Features with ICCs <0.75 were considered nonstable and were eliminated. Pearson’s correlation analysis was used to identify redundant features, and for any two features with a coefficient of 0.9, the one with the larger mean absolute coefficient was eliminated. The least absolute shrinkage and selection operator algorithm (LASSO) was applied to select the most significant predictive parameter from the training cohort, and fivefold cross-validation was used to perform dimensionality reduction. A radiomic signature (Radscore) was calculated using a linear combination of the final selected features weighted by their respective coefficients.

## Statistical analysis

SPSS (version 26.0) and R software (version 4.0.5) were used for statistical analyses. Receiver operating characteristic (ROC) curve analysis was used to evaluate the optimum cutoff value of TSR in discriminating 3-year DM risk based on the maximum Youden index. Heat maps showed the distribution of variables between patients with or without DM within 3 years. The independent DMFS risk factors were determined using Kaplan–Meier curves and Cox regression analysis sequentially based on the data of the training cohort. Statistical significance was set at *P*<0.05. Interobserver variability was assessed using κ statistics for categorical and ranked variables, and ICC for continuous variables.

### Model training and validation

According to the results of Cox regression, a radiomic model (Mr) integrating all independent risk factors except TSR, a TSR model (Mt) integrating all independent risk factors except the Radscore, and a combined radiomic-TSR model (Mrt) incorporating all the independent risk factors were constructed to predict the 3-year DM risk. The discriminative ability of these models was evaluated and compared using ROC curves and the Delong test, respectively. Calibration plots were drawn to explore the calibration ability of the three models. Decision curve analysis was performed to explore the clinical benefits by calculating the net benefit of each decision strategy at each threshold probability.

## Results

### Patients

Patients’ clinicopathological characteristics were evenly distributed between the training and validation groups without significant differences (Table 1). The median follow-up period in the training and validation cohort was 55.3 (52.2–58.4) and 51.8 (44.3–59.2) months, respectively. Among the 302 patients, 58 developed DM within 3 years postoperatively, with 26 in the liver, 20 in the lungs, and 12 in multiple organs. Overall, 35 patients died during follow-up.

**Table 1:** Clinicopathologic characteristics of the patients.

Characteristics	Total (*n*=302)	Training cohort (*n*=211)	Validation cohort (*n*=91)	*P*
Age, years				
Average ± SD	56.1±10.8	56.1±11.0	56.2±10.5	0.928
Sex				
Female	105 (34.8%)	72 (34.1%)	33 (36.3%)	0.720
Male	197 (65.2%)	139 (65.9%)	58 (63.7%)	
BMI, Kg/m^2^				
≥25	160 (53%)	101 (47.9%)	41 (45.1%)	0.653
<25	142 (47%)	110 (52.1%)	50 (54.9%)	
CEA				
Normal	172 (57%)	123 (58.3%)	49 (53.8%)	0.474
Elevated	130 (43%)	88 (41.7%)	42 (46.2%)	
CA19-9				
Normal	194 (64.2%)	138 (65.4%)	56 (61.5%)	0.520
Elevated	108 (35.8%)	73 (34.6%)	35 (38.5%)	
MR-T stage				
2	18 (6%)	10 (4.7%)	8 (8.8%)	0.132
3	243 (80.5%)	176 (83.4%)	67 (73.6%)	
4	41 (13.5%)	25 (11.8%)	16 (17.6%)	
MR-N stage				
0	193 (63.9%)	138 (65.4%)	55 (60.4%)	0.669
1	92 (30.5%)	61 (28.9%)	31 (34.1%)	
2	17 (5.6%)	12 (5.7%)	5 (5.5%)	
ypT stage				
0	21 (7.0%)	15 (7.1%)	6 (6.6%)	0.993
1	23 (7.6%)	17 (8.1%)	6 (6.6%)	
2	80 (26.5%)	55 (26.1%)	25 (27.5%)	
3	168 (55.6%)	117 (55.5%)	51 (56.0%)	
4	10 (3.3%)	7 (3.3%)	3 (3.3%)	
ypN stage				
0	258 (85.4%)	179 (84.8%)	79 (86.8%)	0.655
1	44 (14.6%)	32 (15.2%)	12 (13.2%)	
Adjuvant therapy				
Yes	92 (30.5%)	61 (28.9%)	31 (34.1%)	0.372
No	210 (69.5%)	150 (71.1%)	60 (65.9%)	

CEA, carcinoembryonic antigen; MR-T stage, MRI evaluated T stage; MR-N stage, MRI evaluated N stage; ypT stage, pathological T stage after neoadjuvant chemoradiotherapy; ypN stage, pathological N stage after neoadjuvant chemoradiotherapy; CA19-9, cancer antigen 19-9.

### Tumor stroma assessment and radiomic study

**Figure 2:**
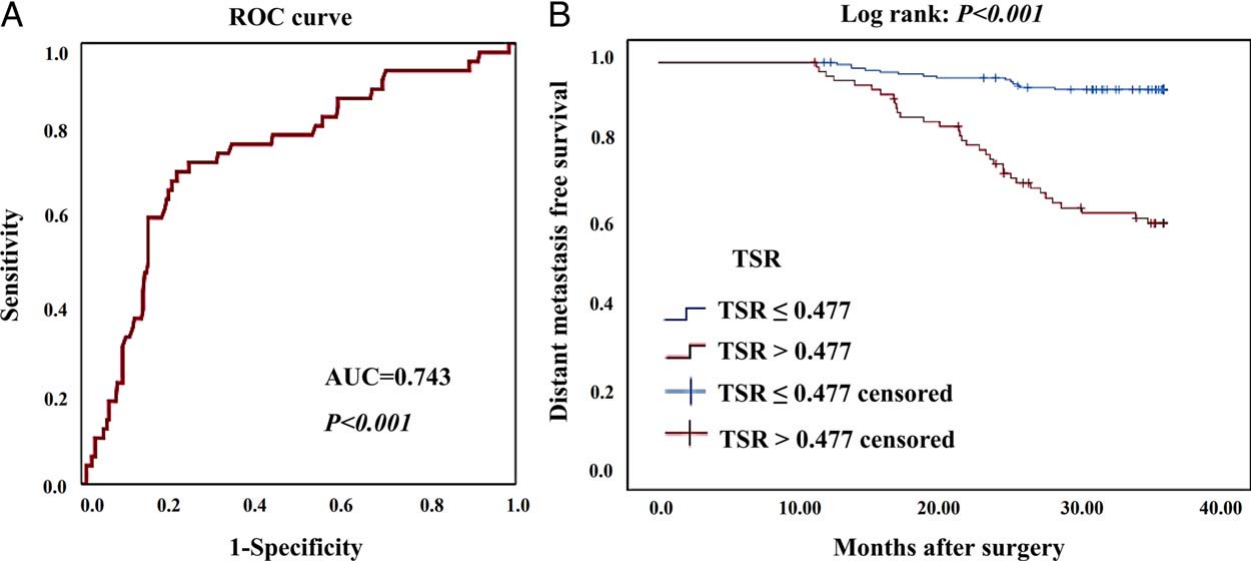
A, ROC curve shows that the TSR exhibited a significant value in predicting the 3-year distant metastases risk in patients with LARC, with an AUC of 0.743 (*P<*0.001). B, Kaplan–Meier curves show that the low-stroma (TSR ≤0.477) group had a significantly better distant metastasis-free survival than the high-stroma (TSR >0.477) group (*P<*0.001). AUC, area under the curve; ROC, receiver operating characteristic; TSR, tumor stroma ratio.

The median TSR assessed using colonoscopic biopsies was 0.424 (0.235–0.684) in the training cohort and 0.414 (0.223–0.667) in the validation cohort. ROC curve analysis revealed that the TSR significantly correlated with the 3-year DM risk, with an area under the curve (AUC) of 0.743 (*P<*0.001, 95% CI: 0.664–0.822) (Fig. 2A), and the optimal cutoff value was 0.477 (Sen=70.8%, Sep=78%). The patients were subsequently stratified into high-stroma (TSR >0.477) and low-stroma (TSR<=0.477) groups. Kaplan–Meier curves (Fig. 2B) showed that the overall 3-year DMFS was significantly higher in the low-stroma group than in the high-stroma group (92.2% vs. 59.3%, *P<0.001*).

**Figure 3:**
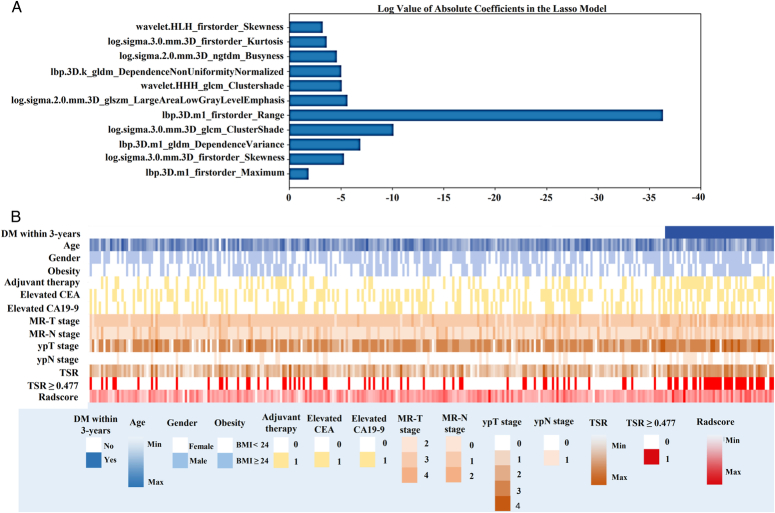
A, Log values of absolute coefficients of 11 selected radiomic features in the least absolute shrinkage and selection operator algorithm model. B, The heat map shows the clinical and MRI features distribution between patients with or without DM within 3 years. Each row in the map corresponds to a unique factor, color-coded as detailed in the legends on the left and below. Each column represents one particular patient. MR-T and MR-N stages, ypT, and ypN stages, TSR (either as a continuous variable or a binary variable grouped by the cutoff value of 0.477), adjuvant therapy, and the Radscore showed different distributions between the two groups. CEA, carcinoembryonic antigen; CA19-9, cancer antigen 19-9; DM, distant metastases; MR-T stage, MRI evaluated T stage; MR-N stage, MRI evaluated N stage; ypT stage, pathological T stage after neoadjuvant chemoradiotherapy; ypN stage, pathological N stage after neoadjuvant chemoradiotherapy; TSR, tumor stromal ratio.

Excellent interobserver consistency was found regarding radiomic features (ICC=0.852–0.923). LASSO regression identified 11 features with nonzero coefficients in the training cohort. Based on these features, the Radscore was constructed using a linear combination (Fig. 3A).

### Model training and validation

Table 2 summarizes the results of Kaplan–Meier analyses and Cox regression of the clinicopathological variables based on the dataset of the training cohort. The TSR (HR=3.072, *P*=0.006), MR-T stage (HR=2.660, *P*=0.023), ypN stage (HR=2.362, *P*=0.028), and Radscore (HR=719.231, *P*=0.023) were proven to be independently correlated with DMFS. The heat map shown in Figure 3B displays the distribution of all variables between patients with or without DM within 3 years: MR-T and MR-N stages, ypT and ypN stages, TSR (either as a continuous variable or a binary variable grouped by the cutoff value of 0.477), adjuvant therapy, and the Radscore show different distributions between the two groups.

**Table 2:** Kaplan–Meier and Cox regression analyses of independent risk factors for DMFS in the training cohort.

Variables	Kaplan–Meier analysis		Cox regression analysis	
	HR (95% CI)	*P*	HR (95% CI)	*P*
Age, years (>56 vs. ≤56)	1.187 (0.626–2.251)	0.599		
Sex	1.121 (0.566–2.222)	0.743		
BMI, Kg/m^2^ (≥25 vs. <25)	1.416 (0.747–2.684)	0.287		
Elevated CA-199	1.753 (0.923–3.328)	0.086		
Elevated CEA	1.872 (0.988–3.550)	0.055		
TSR (≥0.477 vs. <0.477)	6.371 (3.206–12.660)	**<0.001**	3.072 (1.381–6.835)	**0.006**
MR-T stage	5.231 (2.666–10.265)	**<0.001**	2.660 (1.143–6.187)	**0.023**
MR-N stage	1.660 (1.055–2.611)	**0.028**	0.940 (0.054–1.627)	0.826
ypT stage	3.061 (1.706–5.493)	**<0.001**	1.359 (0.777–2.377)	0.283
ypN stage	5.747 (3.000–11.011)	**<0.001**	2.362 (1.100–5.073)	**0.028**
Radscore	19450.692 (88.585–4270787.614)	**<0.001**	719.231 (2.439–212128.935)	**0.023**
Adjuvant therapy	3.626 (1.911–6.879)	**<0.001**	1.661 (0.778–3.547)	0.189

Bold values are statically significant of *P* <0.05.

CA19-9, cancer antigen 19-9; CEA, carcinoembryonic antigen; TSR, tumor stroma ratio; MR-T stage, MRI evaluated T stage; MR-N stage, MRI evaluated N stage; ypT stage, pathological T stage after neoadjuvant chemoradiotherapy; ypN stage, pathological N stage after neoadjuvant chemoradiotherapy.

**Figure 4:**
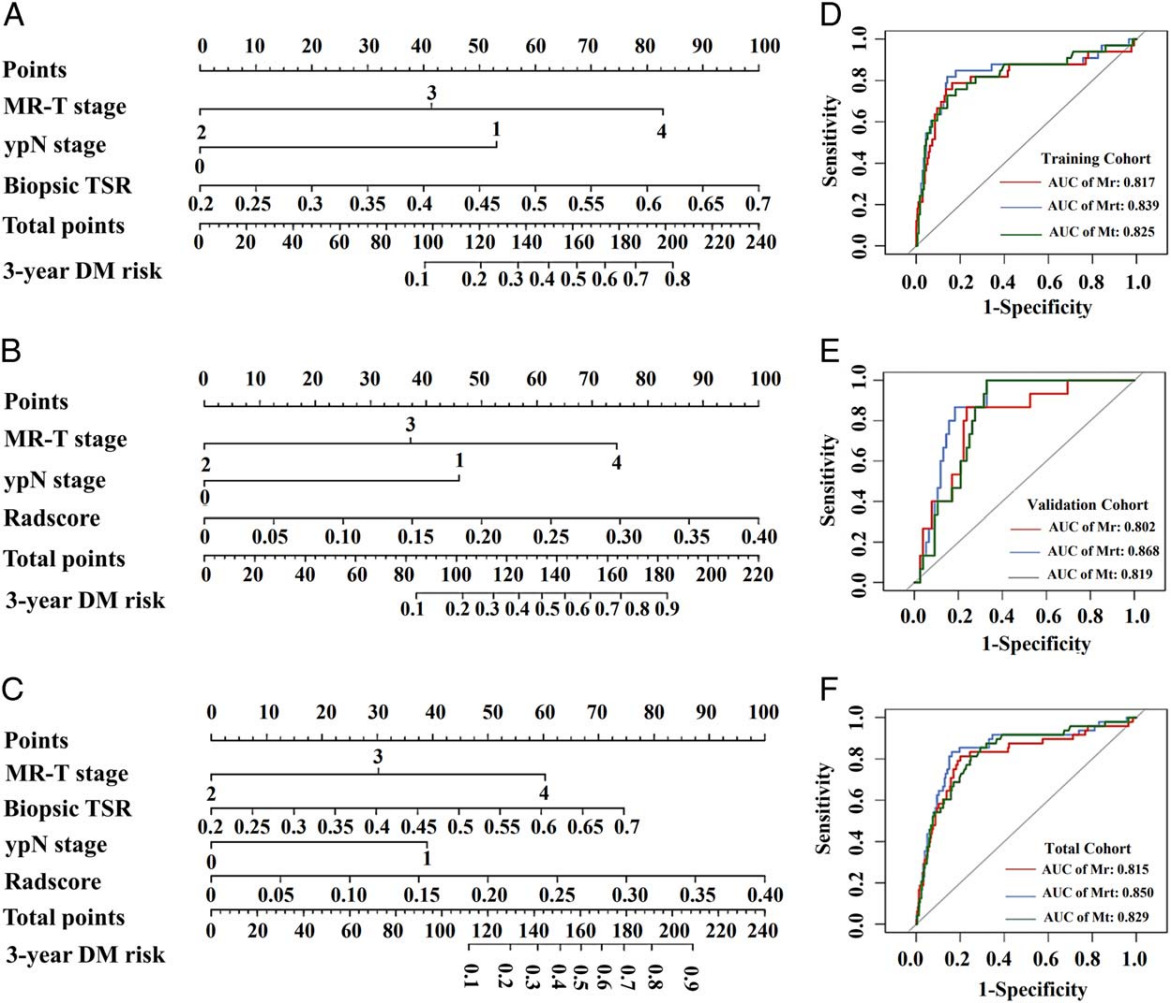
Nomograms (A–C) and receiver operating characteristic curves (D–F) of the three models (Mt, Mr, and Mrt) to estimate the 3-year DM risk in the training, validation, and total cohorts. Mt and Mrt showed slightly improved discrimination performance than Mr. DM, distant metastasis; MR-T stage, MRI evaluated T stage; ypN stage, pathological N stage after neoadjuvant chemoradiotherapy; TSR, tumor stroma ratio; Mr, radiomic model; Mt, TSR model; Mrt, radiomic-TSR model.

**Figure 5:**
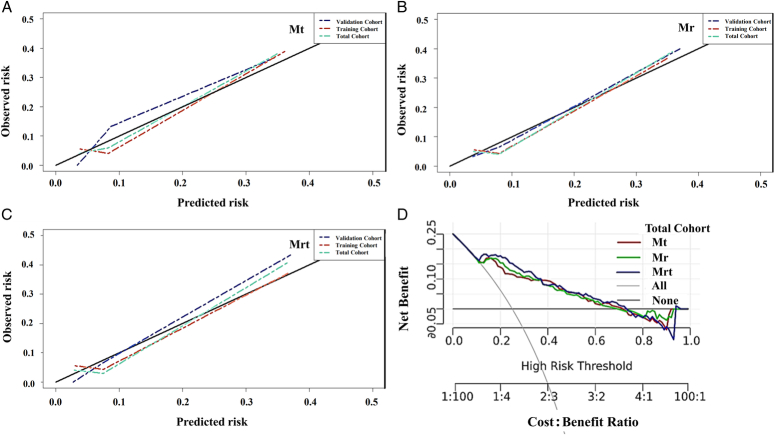
Calibration curves of Mt (A), Mr (B), and Mrt (C) to estimate the 3-year distant metastasis risk in the training, validation, and total cohorts. All the models show excellent predictive stability and accuracy. (D) Decision curves of Mt, Mr, and Mrt to estimate the 3-year distant metastasis risk. Mrt or Mr did not show better benefit gains than Mt. Mr, radiomic model; Mt, TSR model; Mrt, radiomic-TSR model.

Three nomogram models were built to stratify the 3-year DM risk based on part or all of the four independent risk factors. Mt is based on the mrT stage, ypN stage, and TSR (Fig. 4A). Mr was based on the mrT stage, ypN stage, and Radscore (Fig. 4B). Mrt is based on all four factors (Fig. 4C). In the ROC analysis, Mrt had the highest AUCs (0.839, 0.868, and 0.850), followed by Mt (0.825,0.819, and 0.829), and Mr (0.817,0.802, and 0.815) in the training, validation, and total cohorts (Fig. D–F). In the Delong test (Table 3), Mrt performed significantly better than Mr in the validation and total cohorts. However, Mrt did not exhibit a significant advantage over Mt. The calibration curves of the three models are presented in Figure 5A–C, demonstrating excellent prediction accuracy and stability.

**Table 3:** Delong test compared the AUC of the three models in training, validation, and total cohort, respectively.

z (P value)	Training cohort	Validation cohort	Total cohort
Mr vs. Mt	0.47 (*P*=0.641)	0.40 (*P*=0.686)	0.76 (*P*=0.447)
Mrt vs. Mt	0.99 (*P*=0.322)	1.96 (*P*=0.050)	1.72 (*P*=0.086)
Mrt vs. Mr	1.65 (*P*=0.099)	**1.98** (***P*** **=0.048)**	2.49 (***P*** **=0.013)**

Bold values are statically significant of *P* <0.05.

Mr, radiomic model; Mrt, radiomic-TSR model; Mt, TSR model.

### Clinical utility

Decision curve analysis showed that all three models significantly improved the predictive value of 3-year DMFS in patients with LARC. Using Mrt or Mr did not result in better gains than Mt (Fig. 5D).

## Discussion

This study demonstrated that the TSR evaluated from colonoscopic biopsies could independently predict the risk of metachronous DM in LARC patients treated with NCRT and radical surgery. Radiomic signatures from rectal MRI, MR-T stage, and ypN stage were independent risk factors for DMFS. Three models were built to predict the 3-year DM risk based on some or all of the aforementioned factors. By comparing the accuracy, stability, and clinical benefit of the three models, it was found that the comprehensive performance of Mt was slightly better than Mr and no worse than that of the combined Mrt model.

Approximately 50–60% of patients with colorectal cancer could develop DM during the disease, which is the leading cause of treatment failure^6^. Investigations of tumors have mainly focused on neoplastic cells. However, it has increasingly been realized that tumor cells alone cannot fully explain their heterogeneity; the stroma within which the tumor resides and the interaction between the two plays crucial roles in tumor development and progression. The tumor stroma comprises inflammatory and immune cells and extracellular components, such as the matrix, vasculature, and cytokines. These elements can drive tumor cell progression and migration, promote tumor angiogenesis, and block the effects of anticancer agents^13^. In several studies on RC patients, the features of the tumor stroma outperformed TNM staging^14–16^, and the proportion of stromal components significantly affected RC progression^15–17^. On this basis, the TSR scoring system was developed to calculate the amount of stroma in the entire tumor as a ratio^18^, which is an important determinant of RC progression and prognosis^15–17,19–21^, with high reproducibility and feasibility. However, there are still deficiencies in the application of TSR in clinical practice; TSR was conventionally assessed using surgical specimens, which does not apply to RC patients who receive NCRT, because of the dramatic changes in the tumor microenvironment after treatment. Furthermore, the TSR cannot be measured for patients deemed ineligible for surgery. Studies show that the TSR scores of biopsies were highly correlated with matching resection material (81%) in esophageal cancer^22^, and the biopsic TSR can accurately predict the survival outcome of patients with breast cancer^23^ and laryngeal cancer^24^. To our knowledge, no such studies have been conducted on patients with RC.

This study used colonoscopic biopsies to evaluate the TSR and confirmed its value in the stratification of metachronous DM risk in patients with LARC after NCRT and radical excision. According to the Kaplan–Meier analysis, the DMFS in the low-stoma group was significantly better than that in the high-stroma group (92.2% vs. 59.3%, *P<*0.001). In addition, ROC analysis revealed that the optimal cutoff value of biopsic TSR for distinguishing patients with a high DM risk was 0.477. This was consistent with previous studies, where the optimal cutoff value for maximum discriminative power was 0.5^19^. We speculated that the reason for the lower cutoff value of biopsic TSR was that the surgical TSR was determined on the slide(s) with the deepest invasion of the whole tumor, which may have a higher average level of stromal component. As the sampling of biopsy tissue is subject to random variation, ensuring representation of the most aggressive tumor area is not guaranteed, potentially leading to a lower average TSR value. In addition, studies reported that the pathologist-reported TSR tends to be overestimated by ~10%^17^. In contrast, our study adopted an automated TSR scoring method to reduce this subjective bias.

Although colonoscopy necessitates the acquisition of a range of samples from multiple tumor sites, it still fails to capture the overall tumor heterogeneity. This study employed radiographic methods to address these limitations. MRI is crucial in evaluating RC, offering insights into the tumor’s overall heterogeneity and surroundings. Our previous studies^3,25,26^ revealed significant correlations between MRI characteristics of RC and patient survival, tumor staging, and NCRT response. Radiomic features derived from the images were also reported to be closely related to tumor heterogeneity and patient outcomes^27–30^, while their disadvantages have also been highlighted, including a high requirement for image quality, complicated operation processes, unclear biological roles, and pitfalls such as class imbalances and overfitting^8^. This study filtered out 11 MRI radiomic features correlated with patients’ DMFS to construct the Radscore and validated its predictive value for the risk of 3-year DM. By constructing and comparing the performance and clinical-decision-support values of the three models, we demonstrated that a clinical model (Mt) based on the biopsic TSR was as effective as the combined model, and more effective and convenient than the radiomic model (Mr). We recommend the Mt model for stratifying the risk of 3-year DM in LARC patients eligible for colonoscopic biopsy and TSR assessment.

Among the other clinical, radiographic, and pathological factors evaluated in this study, the MR-T and yp-N stages were independently associated with DMFS. Similar results have been reported in previous studies. Li *et al*.^31^ analyzed 148 patients with RC treated with total resection without NCRT or adjuvant therapy and found that the pT stage was independently correlated with DM risk. In our previous multi-center study^28^ including 629 patients with LARC, ~35% received NCRT. The study demonstrated that, apart from the radiomic signature and surgical modalities, the pN stage emerged as the sole independent factor influencing the risk of DM. In this study, because all patients received NCRT preoperatively, pathological T/N staging could not accurately reflect the original state of the tumor. Therefore, we included both the pathology- and MRI-evaluated T and N stages in this study and verified their independent influences on DMFS. The initial T stage of RC represents the overall tumor load and extent of invasion. Consequently, the higher the initial T stage of the tumor, the higher the risk of DM or recurrence. MRI can accurately assess the initial T stage of RC^3^; therefore, in this study, the MR-T stage showed an independent effect on DMFS. However, nodal evaluation using MRI remains challenging^32,33^, which may explain why the MR-N stage failed to show an independent effect on DMFS. In this study, the ypN stage, representing the node status after NCRT, showed an independent effect on DMFS, probably because it can still indicate an advanced local stage and a poor treatment effect.

Overall, adjuvant therapy should be considered as a protective factor against DM; however, in this study, it was a risk factor for DM in the univariate analysis. This is probably because patients who received adjuvant therapy tended to have high-risk pathological factors, such as a more advanced T or N stage, neurovascular invasion, or molecular subtypes correlated with a poor prognosis. All these confounding factors may ultimately contribute to the loss of the independent influence of adjuvant therapy in the multivariate analysis.

Our study had some limitations. First, this retrospective study was conducted at a single center, which may lead to selection bias. Second, the TSR was measured using biopsy specimens, where the biopsy site may have introduced potential influences. Although the TSR of biopsy specimens showed good predictive value for DMFS in our study, external validations in large series are essential. We are going to initiate prospective validation work at our institute and multiple regional medical cancer centers in the near future. If the benefits can be reproduced similarly, we believe the prognostic importance of these approaches would create a significant impact in the clinic. Finally, improvements in the MRI staging of RC, especially in the N staging and post-NCRT evaluation, are mandatory.

In conclusion, we demonstrate that TSR evaluated from colonoscopic biopsies provides significant value in stratifying the DM risk of patients with LARC. We recommend clinicians use TSR biopsy assays, baseline MRI, and surgical pathological examinations to stratify DM risk. In patients unavailable for biopsy TSR evaluation, the prediction can be effectively accomplished through MRI radiomics analysis. These models can be helpful for individualized treatment planning and surveillance.

## Ethical approval

The collection of clinicopathologic data of the subjects was approved by the relevant ethics review board at the National Cancer Center/Cancer Hospital, Chinese Academy of Medical Sciences, and Peking Union Medical College. Approval number 22/449-3651.

## Consent

This study was retrospectively registered and was approved by the review board of our institute, with a waived informed consent (22/449-3651).

## Source of funding

The research was funded by:

1. Youth program of Beijing Natural Science Foundation (7244398).

2. CAMS Innovation Fund for Medical Sciences (CIFMS) (2022-I2M-C&T-B-077 & 2021-I2M-C&T-A-017).

3. Beijing Hope Run Special Fund of Cancer Foundation of China (LC2021A12).

4. Capital’s Funds for Health Improvement and Research (CFH) (2022-2-4024).

5. National Key R&D Program for Young Scientists (SQ2022YFC2500011).

6. National Natural Science Foundation of China (82100598).

The study sponsor was Hongmei Zhang, who make the main contribution in the collection and decision to submit the manuscript for publication.

## Author contribution

Q.Z., H.Z., and X.G.: make equal contribution in study design, data collection, data analysis, and interpretation; Q.Z. and H.Z.: drafted the manuscript; L.W.: performed the model construction; X.Z.: supervised the radiomic analysis; S.Z.: supervised the pathological analysis and co-corresponding author; H.Z.: was the sponsor of the research and corresponding author, conducted research quality control and paper revision. All authors discussed the results, provided critical feedback, and helped shape the research, analysis, and manuscript.

## Conflicts of interest disclosure

The authors declare that they have no financial conflict of interest with regard to the content of this report.

## Research registration unique identifying number (UIN)

NCT06293612.

## Guarantor

Hongmei Zhang.

## Data availability statement

The datasets used and analyzed during the current study are available from the corresponding author on reasonable request.

## Provenance and peer review

Not invited.

## Supplementary Material
