# Supplementary material for: Role of microenvironment characteristics and MRI radiomics in the risk stratification of distant metastases in rectal cancer: a diagnostic study
Source: Int J Surg. 2024 Sep 4;111(1):200–9. doi: 10.1097/JS9.0000000000001916 (PMC11745655; doi:10.1097/JS9.0000000000001916)
Supplement: Supplementary file 2 [file js9-111-0200-s002.docx]

Table1 Protocols for the MRI examination.

| **Sequence** | **TR (ms)** | **TE (ms)** | **FOV (cm)** | **Matrix** | **Bandwidth (kHz)** | **NEX** | **ETL** | **Thickness (mm)** | **gap (mm)** |
| --- | --- | --- | --- | --- | --- | --- | --- | --- | --- |
| **T1-Weighted Image** | 560 | Min | 34 | 288×224 | 41 | 2 | 4 | 5 | 0.5 |
| **Sagittal T2-Weighted Image** | 4800 | 115 | 24 | 256×320 | 41 | 4 | 21 | 4 | 0.4 |
| **Oblique axial T2- Weighted Image** | 4800 | 115 | 16 | 256×320 | 41 | 4 | 21 | 3 | 0 |
| **Fat-saturated axial T2- Weighted Image** | 5700 | 85 | 34 | 288×224 | 31 | 2 | 21 | 5 | 0.5 |
| **Diffusion- Weighted Image (b=0, 1000s/mm^2^)** | 2300 | Min | 34 | 128×160 | 250 | 2 | NA | 5 | 0.5 |

TR, repetition time; TE, echo time; FOV, field of view; NEX, number of excitation; ETL, echo train length
